# The “Little MonSta” Deep-Sea Benthic, Precision Deployable, Multi-Sensor and Sampling Lander Array

**DOI:** 10.3390/s21103355

**Published:** 2021-05-12

**Authors:** Andrew J. Wheeler, Aaron Lim, Felix Butschek, Luke O’Reilly, Kimberley Harris, Paddy O’Driscoll

**Affiliations:** 1 School of Biological, Earth & Environmental Sciences/Environmental Research Institute, Distillery Fields, North Mall Campus, University College Cork, Cork, Ireland; aaron.lim@ucc.ie (A.L.); felix.butschek@ucc.ie (F.B.); luke.oreilly@ucc.ie (L.O.); kimberley.harris@dpenergy.com (K.H.); 2 Irish Centre for Research in Applied Geosciences, University College Cork, Cork, Ireland; 3 Marine & Renewable Energy Institute, University College Cork, Cork, Ireland; 4 Green Rebel Marine, Crosshaven Boatyard, Crosshaven, County Cork, Ireland; 5 DP Energy Ireland Ltd., Mill House, Buttevant, County Cork, Ireland; 6 P&O Maritime Logistics, Parkmore Business Park West, Galway, Ireland; paddy.odriscoll@pomaritime.com

**Keywords:** benthic lander, seabed monitoring, ADCP, sediment trap, cold-water coral, submarine canyon

## Abstract

The “Little MonSta” benthic lander array consists of 8 ROV-deployable (remotely operated vehicle) instrumented lander platforms for monitoring physical and chemical oceanographic properties and particle sampling developed as part of the MMMonKey_Pro program (mapping, modeling, and monitoring key processes and controls in cold-water coral habitats in submarine canyons). The Little MonStas offer flexible solutions to meet the need to monitor marine benthic environments during a historically unprecedented time of climate-driven oceanic change, develop an understanding of meso-scale benthic processes (natural and man-made), and to calibrate geological environmental archives. Equipped with acoustic Doppler current profilers (ADCPs), sediment traps, nylon settlement plates and homing beacons, the compact and upgradable lander platforms can be deployed by ROVs to precise locations in extreme terrains to a water depth of 3000 m. The array allows cluster-monitoring in heterogeneous environments or simultaneous monitoring over wider areas. A proof-of-concept case study was presented from the cold-water coral habitable zone in the upper Porcupine Bank Canyon, where the Little MonStas collected 868.8 h of current speed, direction, temperature, and benthic particulate flux records, as well as 192 particle samples subsequently analyzed for particular organic carbon (POC), lithic sediment, live foraminifera, and microplastics. The potential to upgrade the Little MonStas with additional sensors and acoustic releases offers greater and more flexible operational capabilities.

## 1. Introduction

Oceans are dynamic environments typified by movement, transfer, and change. They are also enigmatic, as their vastness and inhospitality makes them difficult to study. Nevertheless, as the dominant realm on this planet, harboring most life, having a major control of planetary functioning and (biogeo)chemical processes, understanding ocean functioning and its dynamics is vital to managing human interactions with our planetary environment and the sustainability of the ocean’s vast resources. Oceans are the primary regulators of the global climate, acting as an important sink for greenhouse gases and also providing most of the oxygen that we breathe (https://sdgs.un.org, accessed on 10 May 2021). Understanding our oceans has never been more pertinent than now where anthropogenically influenced atmospheric and land-use changes are having a profound effect on our planet’s atmosphere and linked ocean systems [1,2]. For this reason, the United Nations has flagged “Life Below Water” as Goal 14 of the 17 UN Sustainable Development Goals (https://sdgs.un.org/goals, accessed on 10 May 2021). This goal sets out to “conserve and sustainably use the oceans, seas and marine resources for sustainable development”.

Similar to the atmosphere–land interface which humans inhabit, the ocean–seafloor interface is also an important habitat occupied by benthic organisms and visited by nektonic and some planktonic organisms as part of their life cycle. The transfer of particles from the ocean to the seafloor and ocean–seabed chemical reactions are important and pervasive components of ocean functioning and biogeochemical cycles. Seabed mapping and sampling provide valuable spatial information on environmental processes at the ocean–seafloor interface. However, to truly understand these processes, especially in a changing ocean, it is essential to move beyond mapping and spatial sampling to temporal studies. This can be achieved by deploying sensors to collect time-series data [3] or by accessing geological archives by coring the seabed [4]. Understanding ocean change through core analysis has produced impressive results but are limited by sedimentation rates that generate relative low-resolution records covering 100 s to 1000 s of the year. Understanding daily (on the timescale of tides) to annual changes (on seasonal timescales) requires sensor-based monitoring programs. Furthermore, sensors have the capacity to capture the relationship between contemporary variables and thereby calibrate paleo-proxy core data. Ireland possesses an extensive marine territory with a lengthy, embayed continental margin. At mid-latitudes in the NE Atlantic, Ireland’s continental margin covers a transition from a canyon-dominated margin in the south to a glacially-influenced margin in the north [5]. The remoteness of some of the margin limits terrestrial influences, thereby offering the possibility of monitoring background NE Atlantic environmental status. The shelf-detached Rockall and Hatton Banks also provide unique continental margin conditions. Ireland is also one of the few countries to have fully mapped its deep-water continental margin, revealing a variety of features, environments, and habitats [6] including cold-water coral habitats [7,8] which are internationally recognized as significant. Nevertheless, a major challenge exists in exploring this territory in higher resolution and understanding the dynamics of key processes.

This paper outlines the capabilities of a benthic monitoring station or “lander” array designed to provide a temporal monitoring capacity which can be precisely deployed to cover specific spatial coverages even in difficult terrain. The Little MonSta lander array also provides an operational capacity to address Ireland’s desire to reach good environmental status (GES) for its marine water as set out in the EU’s Marine Strategy Framework (EU Directive 2008/56/EC) with a requirement “to establish and implement coordinated monitoring programs for the on-going assessment of the environmental status of marine waters” [9]. Ireland has designated a number of Special Areas of Conservation (SACs) under the EU Habitat’s Directive (EC Directive 92/43/EEC) and is obliged to inspect and monitor their GES. Multi-sensor landers can assist in SAC effective management and develop an understanding of process change beyond assessments of habitat status and impact. Under the EU’s Marine Strategy Framework Directive, Ireland is also developing a series of Marine Protected Areas (MPAs), shifting emphasis away from local habitat conservations to networks of regional conservation areas [10]. Again, multi-sensor landers can assist in providing baseline data to help support and determine effective MPA designation and help meet GES status in Irish waters [9]. On a broader scale, Seabed 2030 represents a coordinated, global effort for mapping the world’s oceans by 2030 [11]. However, given that the ocean is in constant change, even at scales of less than 4 years [12], it is likely that much of the mapped areas will have changed by 2030. Landers such as the Little MonSta array are crucial for understanding how these large-scale, costly datasets will change and to help assess their validity through time.

## 2. Rationale

Most benthic lander systems are single, large multi-sensor platforms that are dropped from the surface vessel to the seabed and recovered using acoustic releases that change the landers’ buoyancy causing the lander to float back to the surface where they are “fished” from the sea [13,14]. These systems have the disadvantage that the deployment area can only be guaranteed within a certain spatial accuracy below the vessel and that smooth landings, especially on uneven seafloors, are not assured. In fact, the precise position and attitude of the lander on the seabed, in some cases, is assumed and positions cannot be adjusted post-deployment. 

The Little MonStas were uniquely designed to meet specific requirements demanded by the MMMonKey_Pro project (http://marinegeology.ucc.ie/mmmonkey_pro, accessed on 10 May 2021); namely, to be precisely deployed on the boundaries of cold-water coral habitats to record environmental variables at the ecosystem tolerances of cold-water coral habitats. As such, the Little MonStas have taken the more novel approach of being designed to be deployable by a remotely operated vehicle (ROV) allowing precision location. Unlike other landers, they are therefore relatively lightweight and compact but nevertheless stable in the strong benthic current regime typical of these habitats. Stability is achieved through the streamline design of the sediment trap housing and the tripod leg configuration. As the Little MonStas do not possess a mooring or acoustic release mechanism for surface recovery, the landers are equipped with a homing beacon to assist in ROV recovery and, in the case of disturbance, a novel innovation with Irish lander deployment.

Most conventional landers carry multiple instrument packages requiring a large frame with wide leg dispersal to ensure stability and record data from one location only. The novel approach of using multiple smaller, compact landers enables a distribution of time-series data points across an area that can be recorded giving a true picture of areal data variability and confidence that data collection is not of only localized significance.

Although initially designed for deep-water deployment in the irregular terrains of a submarine canyon, the Little MonStas can be deployed in a range of settings. The combination of sediment traps/ADCP current profilers and temperature sensors have specific applications such as monitoring sediment plumes in river or estuary settings, or sediment plumes caused by seabed disturbance due to trawling or dredging. Array deployment also allows simultaneous data collection over an area to assess regional benthic conditions (for instance monitoring seasonal particulate flux due to algal blooms or seasonal storm activity) or over an area proposed for development, e.g., a windfarm site.

The Little MonStas have limited instrumentation but become a powerful tool when combined with other datasets. For example, taking Niskin bottle water samples to measure suspended sediment at deployment and recovery calibrates ADCP backscatter data to provide a quantified time-series of suspended sediment within the water column. Similarly, running hull-mounted (downward facing) ADCP profiles during deployment and recovery can add valuable upper water column current data to contextualize higher resolution deeper profiles. Monitoring of benthic current, sediment, and temperature variability may provide useful information on process-drivers for habitat changes documented by repeat seabed mapping including from 3D structure-from-motion photogrammetry of the lander sites. As such, in combination with other data, the Little MonStas can prove to be a powerful tool in understanding benthic environmental dynamics.

## 3. Little MonSta Landers: Sensors and Configuration

**Figure 1:**
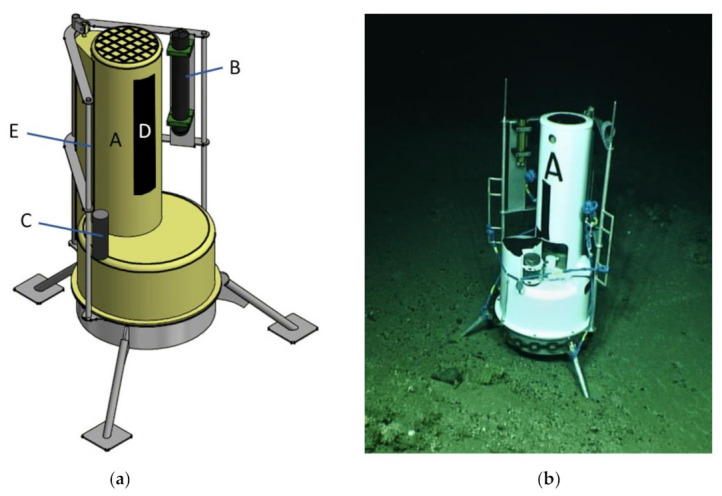
(**a**) Schematic drawing of a Little MonSta and (**b**) deployed with an additional weighted chain on the seabed at 685 m water depth in the Porcupine Bank Canyon. Labeled instruments in the left schematic include: A—Technicap PPS 4/3–24S sediment trap; B—Nortek Aquadopp ADCP profiler; C—Sonardyne homing beacon; D—nylon strip settlement plate; E—ROV manipulator arms grab-holds.

The landers are designed to adapt to the requirements of various surveys with the capacity to change instrument packages and offer a stable platform on the seabed from which data can be collected. The Little MonStas offer this capacity consisting of a Technicap PPS 4/3–24S sediment trap (Technicap, La Turbie, France) (A in Figure 1a) depth-rated to 6000 mwd (meter water depth) in a fiberglass housing (glass-reinforced polyester on an alimentary gel-coat) braced by a marine grade aluminum frame that provides mounting for an array of additional scientific instrumentation as well as ROV manipulator arm grab holds (E in Figure 1a) for ROV-based deployment and recovery.

**Figure 2:**
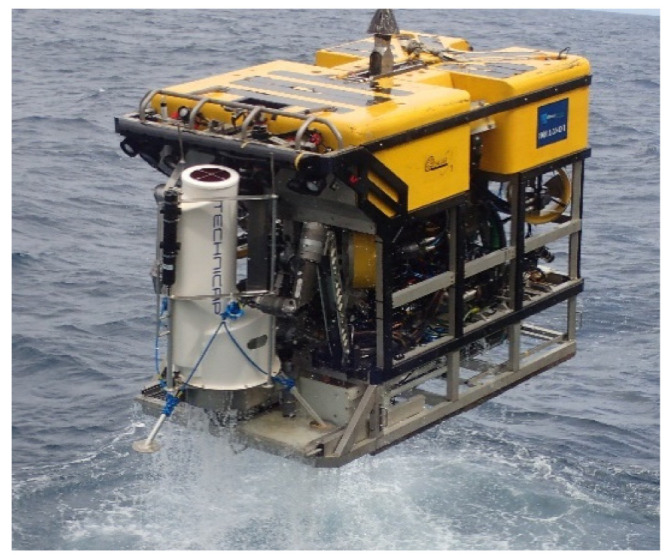
The Holland I ROV recovering a Little MonSta after successful deployment. Note the ROV 7 function manipulator arms clamping the lander to the ROV whilst resting on the ROV chassis. As a fail-safe, the lander is also hooked to the ROV.

The Little MonStas weigh 132.7 kg (in air) and are light enough to be deployed by a work-class ROV (see Figure 2). Additional weights can be added to provide extra seabed stability. A Little MonSta is 1.82 m tall with a tripodal leg arrangement for optimal stability spanning 1.48 m (Figure 1). Footpads are flat with an 18 cm diameter to inhibit sinking into soft sediment, but provide some resistance to lateral movement. In controlled laboratory conditions (flume tank experiment), the Little MonSta can withstand current speeds of up to 1.1 ms^−1^, before sliding across a frictionless surface. In the field, the Little MonStas have maintained position in flows up to 1.14 ms^−1^. Additionally, the ADCP (B in Figure 1a) records tilt, pitch, roll, and heading, thus providing information if and when the Little MonSta moves or falls over.

The teardrop shape of the Technicap sediment trap housing (A in Figure 1a) causes minimal resistance to current flows which reduces sampling bias. Particles (such as sediment, particulate organic matter, and microplastics) settle into the trap at the top of the Little MonSta through a funnel with a 0.05 m^2^ circular collecting area equipped with a 9.5 mm aperture gridded baffle to trap vertical and lateral particulate flows. The cylindro-conical funnel conveys trapped particles to 500 mL sample bottles. A carrousel of 24 bottles is programmed to rotate and open at predefined intervals, using a titanium motor operated on 12-volt alkaline batteries, enabling 18 months of continuous sampling. Prior to deployment, the sediment trap motors can be programmed to sample at a given rate, period, and frequency. All programming is completed within Tera Term version 2.3 (Open Source project https://ttssh2.osdn.jp/index.html.en, accessed on 11 May 2021), a command prompt-style interface which can be connected directly to the motor of the sediment trap. Each of the lander bottles must then be filled with seawater, preferably from the target deployment area to ensure the bottles do not implode when deployed at depth. For longer deployments, a solution of mercury chloride should be added as a “spike” to each bottle to prevent decay of organic material during the deployment. On retrieval of the sediment trap, the data (sample success, operational times) can be accessed directly from the motor via Tera Term v2.3. The samples bottles can then be removed from the lander where they can be capped and stored in a refrigerated space.

Each Little MonSta lander is equipped with a Nortek Aquadopp ADCP Profiler (Nortek, Rud, Norway) (B in Figure 1a) which samples at an acoustic frequency of 1 MHz with an output rate of 1 Hz. Using three slanted beams it measures the Doppler effect [15] which is internally converted to current velocity. The ADCPs can be mounted both vertically and horizontally (Figure 1 shows a vertical mounting), depending on the requirements of the study (e.g., vertical or horizontal current profiles). They have a maximum profiling range of 25 m and a depth-rating of 3000 m. The resolution of the output data is controlled by the cell size which can be set from 0.3 to 4 m. However, this has an impact on the profiling distance. The profiler records velocity and signal amplitude along its three beams for north, east, and vertical directionality with an accuracy of ±1% of the measured value ±0.5 cm s^−1^ up to a current velocity of 10 m s^−1^. Temperature is measured via a thermistor at the ADCP head with an accuracy of 0.1 °C and resolution of 0.01 °C.

The acquisition of ADCP data is controlled by Nortek’s Aquadopp-specific deployment software, Surge. Surge is used to program the sampling rate, cell size, and sampling period, as well as to calibrate the instrument for local magnetic variability. Although sampling rate, period, and duration considerably control battery life, previous usage of the system shows that it can operate from three to nine months [16,17]. The data recorded by the ADCP is stored internally on a local mini-SD card which is only accessible on recovery of the Little MonSta. The data can be read, processed, and visualized via Surge. Subsequently, Surge can export all data (current speed, direction, tilt, roll, heading, and temperature) as a *.csv. Alternatively, the raw data can be loaded into the statistical programming environment R using the “oce” package [18,19].

Nylon strips (D in Figure 1a) are attached to the Little MonStas to measure biofouling quantitatively and qualitatively during the deployment period. The strips of nylon are approximately 5 cm × 30 cm in size and are secured to the lander frame via plastic cable ties. Before deployment, the nylon strips are cleaned with ethanol. On retrieval of the Little MonStas, the strips are photographed, removed whilst wearing gloves, placed in an airlock bag, and refrigerated.

A Sonardyne homing beacon (Sonardyne International Ltd., Yately, UK) (C in Figure 1a) is attached to each Little MonSta frame to expedite lander recovery. This is essential for long deployments when USBL-based locations may be influenced by system recalibrations during the deployment period. The corresponding transducer is mounted on the ROV which, when activated, produces a pulse of acoustic energy between 34 and 40 kHz. When within 750 m of the homing beacon, the x, y, and z offset between the beacon and transducer are measured. The display screen shows a live feed of the distance and bearing to the homing beacon.

## 4. Benthic Environmental Dynamics of the Cold-Water Coral Habitats of the Porcupine Bank Canyon

**Figure 3:**
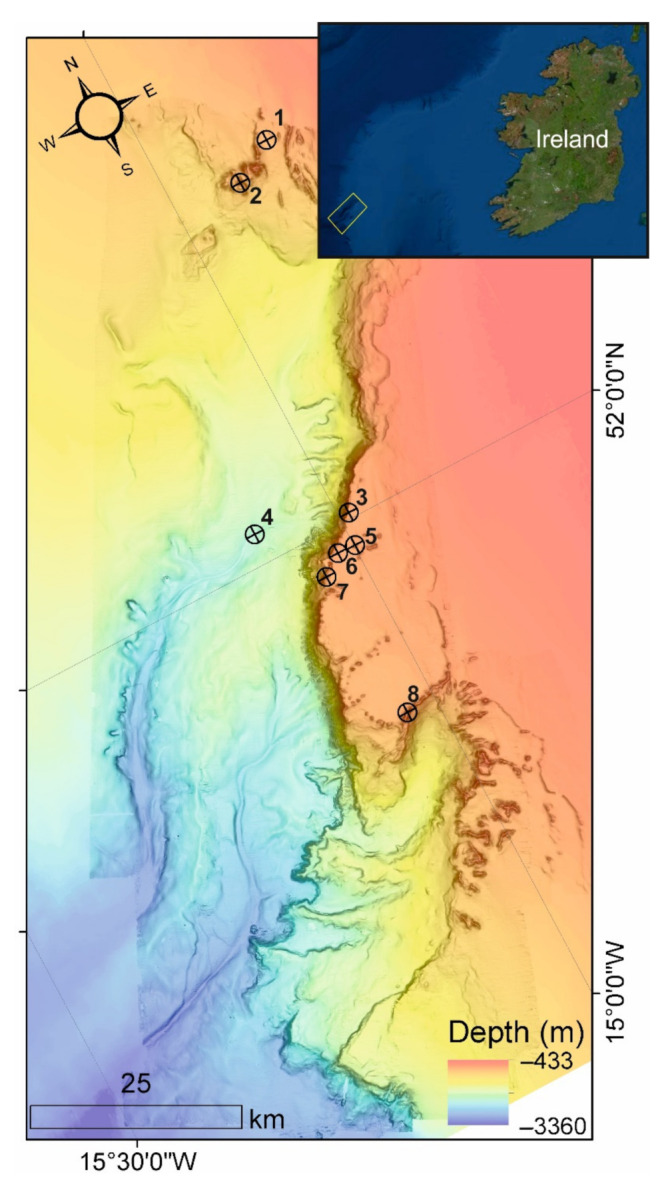
Map of the Porcupine Bank Canyon (main map) and its location on the Western Irish shelf (inset map). Merged bathymetry from newly acquired and existing (lower resolution) data (courtesy of INFOMAR) with Little MonSta positions.

Submarine canyons are complex, geomorphological structures incised into continental margins [20] and act as pathways for sediments and nutrients to the deep-sea funneling particulates from the shelf to the abyssal plain [21]. The Porcupine Bank Canyon (PBC) is one of the largest submarine canyons on the Irish–Atlantic margin (Figure 3) and is designated as an SAC under the EU Habitats Directive hosting a range of cold-water coral habitats [17,22,23,24] within close proximity of one another. These include coral reefs, coral gardens, and coral colonized vertical habitats [23]. The Little MonSta lander array was deployed to determine environmental conditions and dynamics at various locations in the canyon and in particular environmental parameters at the very limits of the cold-water coral habitats. In this way, contemporary conditions could be used to calibrate cold-water coral habitat transitions, cessations, and initiations observed in retrieved cores through coral habitats thereby generating a process-driven understanding of cold-water coral habitat response to changes in the benthic environment through space and time.

In May 2019, 14 ROV dives were completed to survey the PBC and the 8 Little MonStas were deployed: 7 of them at depths between 600 and 840 mwd in the upper PBC and one at 2125 mwd in the deeper canyon (Figure 3) [25]. Deployment operations involved a Little MonSta secured by the ROV (see Figure 2) and deployed vertically to a predesignated destination on the seabed where ROV inspection would fine-tune the precise location. To do this, the live video feed from the ROV could be inspected by experts (scientists and ROV pilots) to make an informed decision on safe and strategic deployment. The Little MonSta was then deployed onto the seabed and the position noted. The immediate surrounds of the site were systematically documented with ROV video after which the ROV was recovered to deck. Deployments averaged 3 h 18 min for the shallower sites and 5 h 25 min for the only deep canyon dive from the time the ROV left the deck to the time it returned. Descent rates ranged from 14 to 24 m min^−1^ with a mean of 18 m min^−1^, while ascent rates averaged 23 m min^−1^. The difference may indicate greater care by the ROV pilot while the lander was attached to the ROV and/or slightly increased drag. During the deployment campaign, two dives were repeated due to hydraulic faults with the ROV and a further two because of the Little MonSta toppled over during deployment, requiring the Little MonStas to be returned to the surface for inspection before redeployment.

The Little MonStas were recovered 2.5 months later [26]. Retrieval operations in the upper PBC carried out in July 2019 were on average 1 h 20 min faster than deployment, with a mean dive time of 1 h 58 min. This was mainly due to fewer video surveys being carried out on ROV dives during the retrieval campaign, though average descent and ascent rates were slightly faster at 24 and 25 m min^−1^, respectively. On average, the Little MonStas were secured to the ROV within 23 min of being spotted on the seabed. The Sonardyne homing beacons were instrumental in speeding up the lander recovery—two landers without homing beacons required an additional 1–2 h of bottom time to find the exact position of the lander.

Six of the Little MonStas each collected more than 9000 valid data points (868.8 h) of current speed, direction, hydrostatic pressure, and temperature. Two of the Little MonSta landers had fallen over during the deployment period, with one recording a maximum flow of 114.2 cm s^−1^ before falling over. While flume tests show that landers can maintain position on a frictionless surface in current speeds of up to 110 cm s^−1^, one of the landers (#7, Figure 3) had fallen over within current speed ranges of 50 to 60 cm s^−1^ after recording nearly 3 days of data on 26 May 2019 at 4:30 a.m. on a muddy, soft, and slightly angled surface. It is unlikely that it had fallen over as a result of the current speed, but that substrate consistency and deployment angle resulted in the lander falling over. Conversely, Lander #3 (Figure 3) fell over after recording for 26 days on 18 June 2019 at 7 a.m. following a period of persistently strong southerly current of 50–80 cm s^−1^. The lander had previously withstood current speeds of 90–114 cm s^−1^ from the south and southwest. However, the peak of currents just prior to the lander toppling over may have been missed if it fell into a 10-min interval between recordings. All other Little MonStas maintained stable positions and recorded full time-series datasets as programmed.

**Figure 4:**
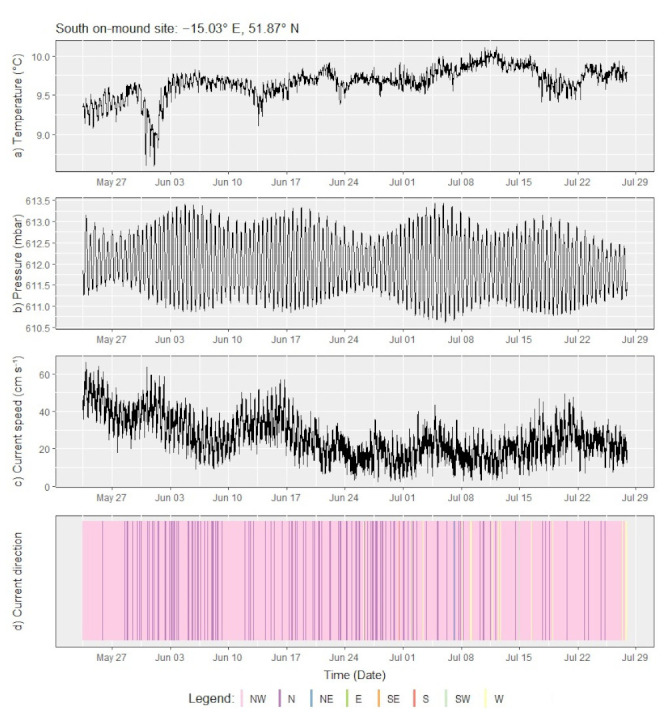
Time series plot of (**a**) water temperature and (**b**) hydrostatic pressure at 1.4 m above the seafloor as well as (**c**) current speed and (**d**) current direction at 3 m above the sea floor with a color legend for the direction on the bottom left. Current speed and direction were measured over a period of one minute in 10-minute measurement intervals, providing >9000 data points per parameter over the deployment period.

Figure 4 shows a time series plot of the measured parameters at various temporal scales. Daily variations in temperature and current speed (Figure 4a,c) correlate to semi-diurnal tidal cycles evident in the pressure record (Figure 4b). At a longer temporal scale, the water temperature dropped by 1.17 °C (from 9.77 to 8.60 °C) over a period of two days from 30 May to 1 June, indicating upwelling of cooler, deeper water from the canyon. Temperatures returned to their pre-cooling average by 3 June. Another two periods of cooling, albeit less pronounced with a drop of only ~0.5–1 °C, can be observed in the temperatures recorded on 13 June and 24 June. These periods of cooling correspond to elevated wind speeds measured at the ocean surface, blowing parallel to the continental slope (either southerly or northerly) and with gusts reaching moderate to fresh gales. Dickson and McCave (1986) [27] attributed near-bed cooling at ~460 mwd to strong northerly (i.e., along-slope) gales, invoking Ekman-transport as the upwelling mechanism on the Porcupine Bank. This earlier observation is corroborated by the findings from the Little MonSta lander array, whereby the canyon may serve as a conduit for Ekman transport-induced upwelling flowing onto the shelf. Finally, the lander array reveals a long-term temporal trend of increasing mean temperatures into the summer months and decreasing current speeds over the sampling period.

The Little MonStas collected 192 particle samples in the sediment traps. A preliminary on-board assessment of the sediment trap contents indicates large variations in sedimentation. Particulate contents ranged from 5–400 mL in volume, with particulate organic matter, suspended muds, silts, sands, and coarse sediments captured in the sample bottles. The largest amount of sediment was trapped in the deep-canyon sampling site, followed by the off-mound site in a channel at the top of the canyon (Figure 3, Landers 4 and 1, respectively), highlighting the role of the canyon as a conduit of particulate flux from the shelf to the deep sea.

The deployment of 8 MonSta landers as an array in strategically placed locations within the canyon thus provides insights into the temporal and spatial dimensions of oceanographic conditions and near-bed processes. Ongoing work investigates benthic-pelagic coupling and mechanisms for how hydrodynamic processes affect coral mound formation and proliferation.

## 5. Comparative Capabilities

Since 1997, nine monitoring sensor deployment campaigns have been undertaken on the Irish continental margin seabed for the purpose of researching cold-water coral habitats involving 25 lander and mooring systems of which the last deployment of the Little MonStas accounts for 32% of systems deployed [17]. Details of the deployments are shown in Table 1 from which capability comparisons with the Little MonStas, top line [17], can be made. Most of the deployments have been on the cold-water coral habitats that have attracted significant scientific research attention.

Table 1 reveals that most of deployments were single or duplication deployments (accounting for 70% of deployments). The two exceptions are with Little MonSta deployment in the Porcupine Bank Canyon outlined above (see previous section and [17]) and the single-sensor deployment of a cluster of ADCP by ROV on the Galway Mound, Porcupine Seabight [29] to record current speed only. This limited investigation [29] and the Little MonSta deployments [17] are the only deployments by ROV facilitating precision locating.

Most of the deployments were at shallow to intermediate water depths (0–1000 m) with only two deployments at an abyssal depths (down to 4500 m) [34]. The Little MonStas were deployed between and 606 and 2125 m water depth [25], though Lim et al. [17] focused their study on the observations from the upper canyon at depths to a maximum of 839 m. It is worth mentioning that the depth range of the studies documented in Table 1 is skewed, given the focus of these studies on cold-water coral habitats which, although not restricted by depth, tend to occur between 600 and 1000 m offshore of Ireland [7]. The maximum deployable water depth is more pertinent with respect to operation capabilities and is limited by the shallowest depth rating of any of the included sensors. As modular systems, all the landers and moorings can be upgraded by swapping out sensors to withstand higher water pressures for deeper deployments. The Little MonStas’ current sensor package is depth rated to 3000 m, although the sediment trap can operate at a depth of up to 6000 m. Information on the maximum deployable depth is available for most of the systems with the Little MonSta providing an average capability.

The duration of deployment mostly depends on the mission objective matching the research question(s). A range of deployment durations have been enacted from >1 to 386 days. The Little MonSta deployment [17] at 76 days sits favorably within this deployment duration range. More pertinent is the maximum endurance time for the deployed systems which is fixed by the battery capacity; however, by reducing the sampling rate on the sensors it is possible to increase battery endurance. There is a direct pay-off between data temporal resolution (sampling rate) and the length of the time monitored with deployment times dependent on the requirements of the research question as well as risk and recovery window constraints. The Little MonStas have an endurance of 365 days comparable with the other systems, although this could be extended with external battery packs.

The different systems are equipped with a range of sensors. All systems recorded current speed except [34,35] (80%), with 40% recording turbidity [3,28,30,32], 40% temperate [3,17,28,32], 30% conductivity (as a proxy for salinity) [3,28,32], 20% for dissolved oxygen [34,35] and 20% fluorescence [30,32]. Five systems (50%) took physical particulate samples [3,17,28,34,35], but only one system (10%) took physical water samples [35]. Unique attribute recording was also undertaken by [32] for benthic photographs, and [17] (the Little MonStas) for biofouling. The most variables measured was performed by the SAMS lander [32] at 6 variables with the average number of variables measured being 3.2 (ranging from 1 to 6). The Little MonStas deployment recorded an above average number of variables at 4 [17].

The novel capabilities of the Little MonStas in comparison with deployed systems on the Irish margin are also highlighted in Table 1. The Little MonStas are the only multi-sensor system that can be precisely located by an ROV and the only system to deploy biofouling tags to abyssal depths.

## 6. Synopsis: Strengths, Weaknesses, and Upgrades

The Little MonSta arrays demonstrate the capacity to collect benthic environmental data in extreme, high current speed, deep marine settings and compares favorably with other systems deployed on the Irish continental margin in its capabilities (Table 1). The key strength of the Little MonStas is their ability to cluster-monitor environments, offering distinct advantages over single point deployments. As such, data from one Little MonSta is confirmed through comparison with data from adjacent stations and site-specific data signals can be isolated. Furthermore, spatial data heterogeneity can be assessed, surmounting the assumption from single point data collections that the data is “typical” of the surrounding area, an assumption that is often not the cases but cannot be quantified.

In addition, the Little MonStas can be precisely located. This offers two strengths: firstly, data can be collected from discrete and highly specific locations. These include small features that would be near impossible to hit with landers dropped from surface vessels, such as small patch reefs, precisely on the summit of channel levees, at the base of steep escarpments or in the middle of pockmarks.

Precision deployment by ROVs also means that the Little MonStas can be deployed safely in areas of extreme topography where surface deployed landers may land at an angle and prove unstable, especially in areas with high currents. The compounding problem with landers that are poorly located is that the consequences of the deployment remain unknown until they are recovered. If landers topple over, they may not release their weights effectively and recovery itself can be compromised. In addition, the entire dataset can be compromised if the landers topple over early. None of these inherent problems with surface deployed landers affect the Little MonStas. Precision deployment and associated reliable data collection opens up a range of seabed terrains for safe and dependable monitoring including topographically extreme environments on seamounts, mid-ocean ridges, slopes, or gullies.

A key weakness with Little MonStas is their dependency on an ROV for deployment and recovery. Although there are advantages to such an approach, not all research vessels are capable of supporting an ROV and access to suitable ROVs may not be readily available in all jurisdictions. Furthermore, the depth of the deployment site is further restricted by the potential maximum depth-rating of the ROV utilized.

A second weakness, perhaps, is the limited sensor array presently available, although this is above average for systems deployed on the Irish margin. Although this can be upgraded (see below), there is a limit to the number of sensors that can be fitted without exceeding the ROV payload. Furthermore, the relatively compact design of the lander, which improves stability, limits unimpeded sensor spacing, a problem not so quickly encountered in wider legged lander platforms.

Increased operability of the Little MonSta array can be enhanced through upgrades. There is limited space for additional sensors with salinometers, pH, and dissolved oxygen (DO) meters as obvious additions. The fitting of acoustic releases activating airbag inflation would reduce the reliance on ROVs. For recovery, this would be cost saving and would facilitate recovery from smaller vessels. Precision deployment by an ROV would still be possible. For missions where precise deployment is not required then the installation of acoustic releases would also enable the addition of greater payloads.

To date, the Little MonSta array has been deployed within cold-water coral habitats [17,25,35], although many other settings could benefit from future deployments. In particular, research questions where spatial as a well as temporal knowledge is required are pertinent. Sediment plume dispersal (from natural or human-induced causes), surface to seabed carbon flux, and regional scale cold-water cascade studies are examples of obvious applications.

## Figures and Tables

**Table 1:** Comparison of lander deployments on the Irish continental margin. The Little MonSta deployment is by Lim et al., 2020 [17].

Publication.	Deployment Mode	Number of Landers	Particulate Samples	Turbidity	Fluorescence	Current Speed	Biofouling	Temperature	Conductivity	Dissolved Oxygen	Water Samples	Photography	Water Depth (m)	Maximum Deployable Depth (m)	Deployment Length (Days)	Endurance (Days)
Lim et al., 2020 [17]	By ROV	8											606–839	3000	76	365
Mienis et al., 2009 [3]	From Vessel	2											554–675	6000	300–330	>365
Mienis et al., 2007 [28]	From Vessel	2											570–281	6000	5–368	>365
Dorschel et al., 2007 [29]	By ROV	6											782–890	#	15–17	#
Duineveld et al., 2007 [30]	From Vessel	1											770	6000	3–291	>365
White et al., 2007 [31]	By Vessel	2											818–870	2000	29–44	365
Roberts et al., 2005 [32]	By Vessel	1											280–842	1200	4–31	#
White, 2003 [33]	From Vessel	2											1000–1200	#	187–297	#
Duineveld et al., 2000 [34]	By Vessel	4											3600–4500	6000	<1	>365
Duineveld et al., 1997 [35]	By Vessel	1											200–4500	#	<1 *	>365

* deployment duration is unclear; # details not specified.

## Data Availability

Not applicable.

## References

[1] Kalnay E., Cai M. (2003). Impact of urbanization and land-use change on climate. Nature.

[2] Dale V.H. (1997). The Relationship Between Land-Use Change and Climate Change. Ecol. Appl..

[3] Mienis F., de Stigter H.C., White M., Duineveld G., de Haas H., van Weering T.C.E. (2007). Hydrodynamic controls on cold-water coral growth and carbonate-mound development at the SW and SE Rockall Trough Margin, NE Atlantic Ocean. Deep. Res. Part I Oceanogr. Res. Pap..

[4] Thierens M., Titschack J., Dorschel B., Huvenne V.A.I., Wheeler A.J., Stuut J.-B., O’Donnell R. (2010). The 2.6 Ma depositional sequence from the Challenger cold-water coral carbonate mound (IODP Exp. 307): Sediment contributors and hydrodynamic palaeo-environments. Mar. Geol..

[5] Wheeler A.J., Devoy R., Cummins V., Brunt B., Bartlett D., Kandrot S. (2021). Ireland’s Continental Margin. The Coastal Atlas of Ireland.

[6] Dorschel B., Wheeler A.J., Monteys X., Verbruggen K. (2010). Atlas of the Deep-Water Seabed.

[7] Wheeler A.J., Beyer A., Freiwald A., de Haas H., Huvenne V.A.I., Kozachenko M., Olu-Le Roy K., Opderbecke J. (2007). Morphology and environment of cold-water coral carbonate mounds on the NW European margin. Int. J. Earth Sci..

[8] Hennige S., Huvenne V.A.I., Meinis F., Wheeler A.J. Waters of the Ireland and the UK. Coral Reefs of the World.

[9] Department of Housing Local Government & Heritage (2020). Marine Strategy Framework Directive 2008/56/EC: Article 17 Update to Ireland’s Marine Strategy Part 1: Assessment (Article 8), Determination of Good Environmental Status (Article 9) and Environmental Targets (Article 10).

[10] Marine Protected Area Advisory Group (2020). Expanding Ireland’s Marine Protected Area Network: A report by the Marine Protected Area Advisory Group. Report for the Department of Housing, Local Government and Heritage, Ireland.

[11] Mayer L., Jakobsson M., Allen G., Dorschel B., Falconer R., Ferrini V., Lamarche G., Snaith H., Weatherall P. (2018). The Nippon Foundation—GEBCO Seabed 2030 Project: The Quest to See the World’s Oceans Completely Mapped by 2030. Geosciences.

[12] Lim A., Kane A., Arnaubec A., Wheeler A.J. (2018). Seabed image acquisition and survey design for cold water coral mound characterisation. Mar. Geol..

[13] Black K.S., Fones G.R., Peppe O.C., Kennedy H.A., Bentaleb I. (2001). An autonomous benthic lander. Cont. Shelf Res..

[14] Santana J.P., Mathias N., Hoveling R., Alves H., Morais T. (2020). Innovative Benthic Lander for Macroalgae Monitoring in Shallow-Water Environments. J. Mar. Sci. Appl..

[15] Seddon N., Bearpark T. (2003). Observation of the Inverse Doppler Effect. Science.

[16] Lim A., O’Reilly L., Summers G., Macedo de Oliveira L., Strachan R. (2020). Holland 1 ROV technical team, Officers and Crew of the RV Celtic Explorer. Systematic Monitoring Survey of the Moira Mound Chain, Cruise Report (CE20011).

[17] Lim A., Wheeler A.J., Price D.M., O’reilly L., Harris K., Conti L. (2020). Influence of benthic currents on cold-water coral habitats: A combined benthic monitoring and 3D photogrammetric investigation. Sci. Rep..

[18] Kelley D.E. (2018). Oceanographic Analysis with R.

[19] Kelley D., Richards C., Layton C. (2021). British Geological Survey *R Package “oce” Documentation*. https://cloud.r-project.org/web/packages/oce/oce.pdf.

[20] Harris P.T., Whiteway T. (2011). Global distribution of large submarine canyons: Geomorphic differences between active and passive continental margins. Mar. Geol..

[21] Liu J.T., Hsu R.T., Hung J.-J., Chang Y.-P., Wang Y.-H., Rendle-Bühring R.H., Lee C.-L., Huh C.-A., Yang R.J. (2016). From the highest to the deepest: The Gaoping River–Gaoping Submarine Canyon dispersal system. Earth-Sci. Rev..

[22] Wheeler A., Capocci R., Crippa L., Connolly N., Hogan R., Lim A., McCarthy E., McGonigle C., O’Donnell E., O’Sullivan K. (2015). QuERCi—Quantifying EnviRonmental Controls on Cold-Water Coral Reef Growth. RV Celtic Explorer, Galway—Moira Mounds—Porcupine Bank Canyon—Galway, 9–23 June 2015. Cruise Report.

[23] Appah J.K.M., Lim A., Harris K., O’Riordan R., O’Reilly L., Wheeler A.J. (2020). Are Non-reef Habitats as Important to Benthic Diversity and Composition as Coral Reef and Rubble Habitats in Submarine Canyons? Analysis of Controls on Benthic Megafauna Distribution in the Porcupine Bank Canyon, NE Atlantic. Front. Mar. Sci..

[24] Mazzini A., Akhmetzhanov A., Monteys X., Ivanov M. (2012). The Porcupine Bank Canyon coral mounds: Oceanographic and topographic steering of deep-water carbonate mound development and associated phosphatic deposition. Geo-Mar. Lett..

[25] Lim A., O’Reilly L., Summer G., Harris K., Shine A., Harman L., Appah J., Macedo de Oliveira L., Boyd J., Anders B. (2019). CE19008 Cruise Report: Monitoring Changes in Submarine Canyon Coral Habitats-Leg 1 (MoCha_SCan).

[26] Lim A., O’Reilly L., Summers G., Harris K., Macedo de Oliveira L., O’ Hanlon Z., Appah A., O’ Mahony E., Strachan R., Walsh P. (2019). CE19014—Monitoring Changes in Submarine Canyon Coral Habitats—Leg 2 (MoCha_ Scan II).

[27] Dickson R.R., McCave I.N. (1986). Nepheloid layers on the continental slope west of Porcupine Bank. Deep Sea Res..

[28] Mienis F., de Stigter H.C., de Haas H., van Weering T.C.E. (2009). Near-bed particle deposition and resuspension in a cold-water coral mound area at the Southwest Rockall Trough margin, NE Atlantic. Deep. Res. Part I Oceanogr. Res. Pap..

[29] Dorschel B., Hebbeln D., Foubert A., White M., Wheeler A.J. (2007). Hydrodynamics and cold-water coral facies distribution related to recent sedimentary processes at Galway Mound west of Ireland. Mar. Geol..

[30] Duineveld G.C.A., Lavaleye M.S.S., Bergman M.J.N., De Stigter H., Mienis F. (2007). Trophic structure of a cold-water coral mound community (Rockall Bank, NE Atlantic) in relation to the near-bottom particle supply and current regime. Bull. Mar. Sci..

[31] White M., Roberts J.M., van Weering T. (2007). Do bottom-intensified diurnal tidal currents shape the alignment of carbonate mounds in the NE Atlantic?. Geo-Mar. Lett..

[32] Roberts J.M., Peppe O.C., Dodds L.A., Mercer D.J., Thomson W.T., Gage J.D., Meldrum D.T. (2006). Monitoring environmental variability around cold-water coral reefs: The use of a benthic photolander and the potential of seafloor observatories. Cold-Water Corals and Ecosystems.

[33] White M. (2003). Comparison of near seabed currents at two locations in the Porcupine Sea Bight—implications for benthic fauna. J. Mar. Biol. Assoc. UK.

[34] Duineveld G., Lavaleye M., Berghuis E., de Wilde P. (2000). Activity and composition of the benthic fauna in the Whittard Canyon and adjacent continental slope (NE Atlantic). Oceanol. Acta.

[35] Duineveld G.C.A., Lavaleye M.S.S., Berghuis E.M., de Wilde P.A.W.J., van der Weele J., Kok A., Batten S.D., de Leeuw J.W. (1997). Patterns of benthic fauna and benthic respiration on the celtic continental margin in relation to the distribution of phytodetritus. Int. Rev. Gesamten Hydrobiol. Hydrogr..

